# A Comparative Clinical Performance of Polymethyl Methacrylate (PMMA) and Urethane Dimethacrylate (UDMA) Materials in Provisional Fixed Prostheses

**DOI:** 10.7759/cureus.63455

**Published:** 2024-06-29

**Authors:** Laith Barqawi, Shaza Kanot

**Affiliations:** 1 Department of Fixed Prosthodontics, Faculty of Dental Medicine, Damascus University, Damascus, SYR

**Keywords:** fixed dental prostheses, crown, resin, udma, pmma, fixed interim restoration

## Abstract

Aim: This study aimed to compare the clinical outcomes of polymethyl methacrylate (PMMA) and urethane dimethacrylate (UDMA) as materials for fixed provisional restorations, focusing on handling properties, chair time, and periodontal outcomes, due to their prevalent use in dental practice.

Materials and methods: A comparative clinical study was conducted with 150 patients at the Department of Fixed Prosthodontics, Damascus University, Damascus, Syria. Patients undergoing prosthetic treatments with crowns and bridges received two fixed provisional restorations using a direct approach. The first, made immediately after abutment preparation, used PMMA. The second, created post-clinical try-in of the final restoration, utilized UDMA. Both restorations were maintained for one week. We assessed chair time, handling properties via the Visual Analog Scale (VAS), and periodontal health using the Plaque Index and Gingival Index. The Kolmogorov-Smirnov test was used to assess data normality. Differences between the two groups in the outcome variables were analyzed using the Mann-Whitney U test. The level of significance was set at (P < 0.05).

Results: The handling properties of chemically activated PMMA resin were superior to those of light-activated UDMA resin. However, UDMA resin outperformed in terms of chair time and periodontal outcomes. The mean chair time was 9.45 ± 1.01 minutes for PMMA and 4.40 ± 0.77 minutes for UDMA. Mild gingivitis or plaque accumulation was observed in 57.3% of PMMA restorations and 44.0% of UDMA restorations. Moderate gingivitis and plaque accumulation were noted in 42.7% of PMMA restorations, while 56.0% of UDMA restorations showed no plaque accumulation or gingival inflammation.

Conclusion: Chemically activated PMMA resin offers excellent handling properties, whereas light-activated UDMA resin provides advantages in chair time and periodontal health, making it a preferable choice for provisional restorations.

Limitations and future research: The study was limited to short-term outcomes and did not assess the long-term durability of the restorations or their aesthetic impact on patient satisfaction. Further studies are recommended to evaluate the long-term performance of these materials, their cost-effectiveness, and their aesthetic outcomes to provide a more comprehensive understanding of their clinical utility.

## Introduction

Despite the rapid evolution of oral health in most countries in the world, fixed prosthodontics is still a widely used treatment option for missing teeth. The conventional approach to fabricating fixed prosthodontic restorations consists of tooth preparation, impression taking, and the fabrication and temporary cementation of a provisional restoration, either directly or indirectly utilizing computer-aided design and computer-aided manufacturing (CAD/CAM) technology. Finally, the dental lab fabricates the final restoration, which is then permanently cemented onto the prepared abutments [1]. Fixed provisional restorations play an important role in the success of the prosthodontic treatment, serving as a valuable diagnostic tool that allows clinicians to continuously evaluate the treatment plan; they are designed to closely resemble the function and the esthetic properties of the final fixed restoration [2]. Provisional restoration fabrication materials can be classified into five categories: chemically activated auto-polymerizing acrylic resins, heat-activated acrylic resins, light-activated acrylic resins, dual light and chemically activated acrylic resins, and alloys. Chemically activated acrylic resins, particularly polymethyl methacrylate (PMMA), are the most common materials used to fabricate fixed provisional restorations [3]. Numerous studies have investigated the properties of PMMA and light-activated acrylic resins, including urethane dimethacrylate (UDMA). The review by Rakhshan 2015 [4] compares the marginal fit of multiple provisional restoration materials and concludes that no material demonstrates superior marginal integrity after long-term use. Idrissi et al. [5] conducted an in vitro study to evaluate the flexural strength of four provisional restoration materials: cold-polymerized PMMA, heat-polymerized PMMA, auto-polymerized bis-acryl composite, and light-polymerized UDMA resin. They concluded that there was no significant difference in the flexural strengths of cold PMMA, hot PMMA, and auto bis-acryl composite; however, the flexural strength of light-polymerized UDMA resin was significantly lower than that of the others. Although numerous papers have discussed the properties of provisional restoration materials, the literature lacks clinical studies that compare the clinical outcomes of PMMA and UDMA as materials for fixed prosthodontic restorations. Addressing this gap, this study evaluates the clinical performance of PMMA and UDMA in fixed provisional restorations, focusing on handling, chair time efficiency, and periodontal health, to inform material selection in clinical practice. The research null hypothesis posits that PMMA and UDMA exhibit equivalent handling properties, chair time, and periodontal outcomes.

## Materials and methods

Between May 2022 and July 2023, we conducted a comparative clinical trial (CCT) on a cohort of 150 patients at the Department of Fixed Prosthodontics, Damascus University, Damascus, Syria. The patients were provided prosthetic treatments and were subsequently included in this study. The inclusion criteria encompassed individuals aged 18 years or older, requiring fixed prosthetic treatments (crowns) within the anterior or posterior regions of either dental arch, with natural dentition opposing the prepared abutments, and demonstrating satisfactory oral hygiene, quantified by an O’Leary plaque index score of less than 40%. We excluded patients who required post and core rehabilitation, those diagnosed with bruxism or untreated periodontal disease, as well as individuals with tobacco usage and frequent soft drink consumption. The integrity of our study’s findings is underpinned by a meticulous patient selection process that adhered strictly to the predefined inclusion and exclusion criteria. We ensured a systematic approach by employing a randomized selection method from the pool of eligible patients who met the study’s requirements. This method was designed to prevent any selection bias, providing an equal opportunity for all qualifying patients to participate. Our team was vigilant in maintaining objectivity throughout the process, and we took additional measures to control for potential confounders. By doing so, we aimed to create a representative sample that accurately reflects the broader patient population, thereby bolstering the validity and applicability of our study’s results. All the treatments were administered to the patients by experienced dentists specializing in fixed prosthodontics at Damascus University. Following abutment preparation, all patients received two consecutive fixed provisional restorations, both fabricated using the direct approach. The first provisional restoration, made immediately after tooth preparation, utilized chemically activated auto-polymerizing acrylic resin PMMA (B&E Crown, B&E Korea Co., Ltd., Korea), employing the silicone impression technique where an impression of the tooth is taken prior to abutment preparation. The second provisional restoration was created post-clinical try-in of the final restoration, using light-activated acrylic resin UDMA (Ranscen Temp C&B LC, Nexobio Korea Co., Ltd., Korea), through the molding technique involving a pre-formed vacuum mold based on a cast model. Both provisional restorations were made, adjusted, and finished in accordance with the manufacturer’s instructions. The restorations were finally temporarily cemented onto the prepared abutments using Eugenol-free temporary zinc oxide-based cement (Provicol QM, VOCO, Cuxhaven, Germany). Following the clinical protocol, both provisional restorations were maintained in the patients' mouths for a duration of one week. This interval allowed for the fabrication of the metal-ceramic fixed prosthodontic restoration following the clinical try-in of the metal core.

This study’s methodology, which involved testing both types of provisional restorations on the same patient, ensures the reliability of the measurements and facilitates a direct comparison under consistent conditions. Chair time, quantified in minutes, was documented for the fabrication of provisional restorations. For the initial provisional restoration, the duration was recorded commencing immediately after the dentist finished the tooth preparation and concluding upon the completion of the provisional restoration’s fabrication. Subsequently, for the second provisional restoration, the timeframe spanned from the completion of the clinical try-in to the finalization of the provisional restoration’s fabrication. Visual Analog Scale (VAS) was utilized to assess the handling properties and ease of use of both materials, with zero indicating utmost ease and 100 indicating extreme difficulty. In the context of our study, “handling properties” refer to the ease of manipulation and application of the provisional restoration materials by clinicians. This encompasses the materials’ consistency, workability, setting time, and overall manageability during the fabrication and placement of the provisional restorations. The periodontal outcomes of the provisional restorations included the Plaque Index (PI) and Gingival Index (GI). Plaque accumulation on the surfaces of provisional restorations was assessed using a UNC-15 periodontal probe (Hu-Friedy Mfg. Co., LLC., Chicago, USA). Each restoration received a PI score from 0 to 3, based on the criteria established by O’Leary et al.: a score of 0 indicated no detectable plaque on any tooth within the segment; 1 denoted a minimal plaque presence not exceeding 2 mm from the gingival margin on any tooth within the segment; 2 signified plaque covering up to half of the exposed clinical crown on any tooth within the segment; and 3 represented plaque extending over more than one-third of the clinical crown [6]. The gingival condition was assessed for all provisional restorations and a score ranging from 0 to 3 was given to each restoration, corresponding to the GI: a score of 0 indicated normal gingiva; 1 denoted mild inflammation - slight change in color and slight edema but no bleeding on probing; 2 signified moderate inflammation - redness, edema and glazing, bleeding on probing; 3 represented severe inflammation - marked redness and edema, ulceration with tendency to spontaneous bleeding [7].

Both indexes were assessed one week after the cementation of the provisional restorations. The evaluation of periodontal outcomes was conducted by three independent dental practitioners to mitigate potential bias. In our study, the periodontal outcomes were meticulously assessed one week after the fabrication of the provisional restorations for both PMMA and UDMA groups. This specific follow-up period was chosen to ensure consistency in the evaluation timeframe, thereby minimizing variability between the two groups. This short-term assessment allowed us to capture the immediate periodontal response to each material, providing a controlled comparison of their respective impacts on gingival health. Future studies may extend this follow-up period to observe long-term periodontal outcomes, which could offer further insights into the sustained effects of these materials over time.

This study was set at the Department of Fixed Prosthodontics, Faculty of Dentistry, Damascus University, and was conducted in accordance with the ethical guidelines of the Helsinki Declaration as revised in 2000. All eligible patients were given the necessary information about the procedure, its purpose, and any potential complications of the procedure materials. In addition, written consent was obtained from all the patients. The protocol of this study was ethically approved by the Ethics Committee of Damascus University (UDDS‐28066022/SRC‐2302).

The sample size for this study was determined using G*Power software (version 3.1.2, Heinrich-Heine-Universität Düsseldorf, Düsseldorf, Germany). This calculation was based on a pilot study of 10 patients (three males and seven females, aged 22 to 56 years), each with one abutment. The study assessed chemically activated PMMA resin and light-activated UDMA resin provisional restorations. Clinicians evaluated the handling properties and ease of use using the VAS. The mean value for chemically activated PMMA resin restorations was 69.20 ± 12.48, while for light-activated UDMA resin restorations, it was 27.30 ± 12.36. We anticipated an effect size of 1.73, which reflects a large expected difference between the groups. To achieve a Type I error rate (alpha level) of 5%, and to ensure a power (1 - beta) of 80%, which is the standard for clinical studies, we calculated that a sample size of 150 patients would be sufficient. This sample size allows us to confidently detect the expected effect while controlling for the possibility of Type II errors. By adhering to these parameters, we aimed to ensure that our study was adequately powered to yield reliable and valid conclusions. Throughout the duration of our study, we maintained a rigorous follow-up protocol to ensure participant retention. We are pleased to report that there were no dropouts during the study period. This was achieved through careful scheduling, consistent communication, and by providing participants with clear instructions and expectations. The absence of dropouts allowed for a complete analysis of all recruited subjects, thereby enhancing the reliability and validity of our study’s results.

The data on participant age, handling properties, and chair time were presented as mean ± standard deviation. In contrast, periodontal outcomes data were expressed as percentages and rank means. The Kolmogorov-Smirnov test was used to assess data normality. Differences between chemically activated PMMA resin and light-activated UDMA resin in the outcome variables were analyzed using the Mann-Whitney U test. The level of significance was set at (P < 0.05). All statistical analyses were performed using SPSS software (IBM SPSS Statistics 29.0, IBM Corp., Armonk, NY, USA).

## Results

Data for 150 patients who received prosthetic treatments were recorded. Each patient received two provisional restorations at two different times. In total, 300 fixed provisional restorations were included in the analysis. These 300 provisional restorations were fabricated for 150 prepared abutments in the maxillary or mandibular arch, either in the posterior or anterior region. Specifically, there were 83 abutments in the upper arch and 67 abutments in the lower arch. Additionally, 100 abutments were located in the anterior region, while 50 abutments were in the posterior region. The majority of the participants were male (54.7%). The mean age of the participants was 39.2 years (SD = ±11.2). The age difference between males (38.6 years, SD = ±11.3) and females (39.8 years, SD = ±11.2) was not statistically significant (p = 0.171).

Handling properties and ease of use

There was a statistically significant difference between chemically activated PMMA resin and light-activated UDMA resin (P < 0.05). Specifically, the chemically activated PMMA exhibited higher handling properties and ease of use. These findings suggest that the chemically activated PMMA resin has better handling properties and ease of use compared to the light-activated UDMA resin. Table 1 demonstrates the mean values and SD for both provisional restorations, along with the results of the Mann-Whitney U test.

**Table 1:** Descriptive statistics and results of Mann-Whitney U test of handling properties PMMA: polymethyl methacrylate; PR: provisional restoration; SD: standard deviation; UDMA: urethane dimethacrylate; * P < 0.05

PR type	Mean ± SD	Lower value	Upper value	Difference between the two means	U value	P
PMMA	26.19 ± 11.5	8	51	-43. 94	0	0.000*
UDMA	70.13 ± 9.71	57	92			

Chair time

The mean value for the chemically activated PMMA resin was 9.45 (SD = ±1.01), while the mean value for the light-activated UDMA resin was 4.40 (SD = ±0.77). These results suggest that the light-activated UDMA resin had a shorter chair time compared to the chemically activated PMMA resin. Furthermore, the Mann-Whitney U test revealed a statistically significant difference between the two groups (P < 0.05), as indicated in Table 2.

**Table 2:** Descriptive statistics and results of Mann-Whitney U test of chair time PMMA: polymethyl methacrylate; PR: provisional restoration; SD: standard deviation; UDMA: urethane dimethacrylate; * P < 0.05

PR type	Mean ± SD	Lower value	Upper value	Difference between the two means	U value	P
PMMA	9.45 ± 1.01	6.35	11.95	-5.06	0	0.000*
UDMA	4.40 ± 0.77	2.32	5.35			

Periodontal outcomes

The accumulation of plaque on chemically activated PMMA resin restorations ranged from mild (57.3%) to moderate (42.7%). In contrast, light-activated UDMA resin restorations showed no plaque accumulation in 56.0% of cases and mild accumulation in 44.0%. The PI rank mean for chemically activated PMMA resin was 206.58, while for light-activated UDMA resin, it was 94.42. Regarding the GI, mild gingivitis was observed in 57.3% of chemically activated PMMA resin restorations and in 44.0% of light-activated UDMA resin restorations. Moderate gingivitis was present in approximately 42.7% of chemically activated PMMA resin restorations. Notably, no gingival inflammation occurred in 56.0% of the light-activated UDMA resin restorations. The GI rank mean for chemically activated PMMA resin was 206.58, and for light-activated UDMA resin, it was 94.42. Consequently, a statistically significant difference existed in terms of plaque and gingival indices between the two resins, with the latter showing better periodontal outcomes (P < 0.05). Refer to Table 3 for the Mann-Whitney U test results.

**Table 3:** Mann-Whitney U results of periodontal outcomes PMMA: polymethyl methacrylate; PR: provisional restoration; UDMA: urethane dimethacrylate; * P < 0.05

Index	PR type	Rank mean	U value	P
Plaque Index	PMMA	206.58	2838.0	0.000*
	UDMA	94.42		
Gingival Index	PMMA	206.58	2838.0	0.000*
	UDMA	94.42		

## Discussion

This study was designed to investigate the clinical outcomes, handling properties, and chair time associated with two provisional restoration materials employed in a direct fabrication approach: chemically activated auto-polymerizing acrylic resin PMMA and light-activated acrylic resin UDMA. To the best of our knowledge, our study is among the few that have evaluated the use of these two provisional restoration materials in terms of handling properties, chair time, and clinical outcomes in such a large sample size.

In the realm of provisional dental restorations, polymeric resins are the materials of choice, predominantly consisting of acrylic and composite resins [8]. The inception of PMMA dates back to 1877, marking a significant milestone in dental material science. It was not until 1937 that PMMA’s application in dentistry was realized, with its initial use in the fabrication of complete denture bases. Subsequently, its utilization expanded into fixed prosthodontics, initially for permanent restorations, and later, in the form of self-curable PMMA, for provisional fixed prostheses. To this day, thermoplastic acrylic resins such as PMMA remain the preferred materials for crafting temporary restorations, owing to their enduring popularity and efficacy [4,9,10]. In scholarly discourse, Bis-phenol A glycidyl methacrylate (Bis-GMA) is recognized as a difunctional monomer with a substantial molecular weight [11]. This monomer’s polymerized variant, when amalgamated with inert filler particles, distinguished itself as the inaugural resin composite in dental applications [4,9]. Progressive enhancements in the molecular architecture of Bis-GMA and its filler integration have catalyzed the emergence of novel compounds, including ethoxylated Bis-GMA, triethyleneglycol dimethacrylate (TEGDMA), and UDMA [12-14]. These advancements have extended the application of these materials beyond their initial scope, with Bis-acryl resins gaining prominence in the production of provisional fixed restorations [4,9].

Our findings revealed significant differences between the two materials in terms of handling properties, chair time, and periodontal outcomes. The chemically activated PMMA resin demonstrated superior handling properties and ease of use, which is consistent with previous research indicating that chemically activated resins generally offer better workability and setting times [15]. This could be attributed to the controlled polymerization mechanisms involved in chemical activation [16], which may provide clinicians with more predictability during the application process that may provide clinicians with more control. In terms of chair time, the light-activated UDMA resin had a significantly shorter duration compared to the chemically activated PMMA resin. This reduction in chair time not only enhances patient comfort but also increases clinical efficiency, allowing for a greater number of patients to be treated within the same timeframe [17]. This could be attributed to the advanced polymerization mechanisms involved in light-activation [18]. Periodontal outcomes also favored the light-activated UDMA resin, with a notable absence of plaque accumulation in a majority of cases. This is a critical finding, as provisional restorations with poor periodontal outcomes can lead to complications such as gingivitis or periodontitis [15]. The lower PI and GI associated with light-activated UDMA resin suggest that it may contribute to better overall oral health during the provisional phase. It is important to note that while the handling properties and chair time are directly controlled by the material properties, periodontal outcomes are also influenced by patient factors such as oral hygiene practices [19]. However, the statistically significant difference in periodontal indices suggests that the material itself plays a pivotal role in minimizing plaque accumulation and gingival inflammation.

Studies such as Stawarczyk et al. have explored the retention strength of PMMA/UDMA-based crowns, which can be indicative of the handling properties of these materials [20]. Additionally, a systematic review and meta-analysis by Astudillo-Rubio et al. assessed the mechanical properties of dimethacrylates and monomethacrylates used in fabricating direct provisional restorations, providing a broader context for comparing PMMA and UDMA [8]. These studies, along with our findings, suggest that light-activated UDMA resin may be the preferred choice for fixed provisional restorations due to its reduced chair time, and favorable periodontal outcomes.

The aesthetic appeal of provisional restorations is a critical factor that significantly impacts patient satisfaction and overall treatment acceptance [21,22]. While our study focused on the clinical performance, chair time, and periodontal health associated with PMMA and UDMA resins, we recognize the importance of the aesthetic dimension in prosthodontic treatments. Therefore, we recommend that future research endeavors include a comprehensive evaluation of the aesthetic outcomes of provisional restorations. Such assessments could involve patient surveys to gauge satisfaction with the appearance of restorations, as well as practitioner evaluations of the restorations’ color match, surface texture, and anatomical form. Although our study has concluded, these recommendations aim to guide subsequent research toward a holistic approach that encompasses both functional and aesthetic considerations in the assessment of provisional dental materials. While our study provides valuable insights into the immediate clinical performance of PMMA and UDMA resins, we acknowledge the importance of understanding their long-term durability. Therefore, we recommend that future research be conducted to assess the longevity and performance of these materials over extended periods. Such studies should focus on the wear resistance, color stability, and structural integrity of the provisional restorations under various oral conditions. This will not only enhance the clinical relevance of our findings but also guide practitioners in selecting materials that ensure the best long-term outcomes for their patients.

This investigation encompassed teeth that had undergone endodontic therapy, thereby limiting the ability to evaluate the influence of provisional restorations on dental sensitivity. Consequently, future research could concentrate on detailed examinations of the efficacy of fixed provisional restorations within distinct clinical environments. Long-term studies are needed to confirm our findings and to explore the cost-effectiveness of using light-activated UDMA resin in various clinical scenarios.

## Conclusions

Our comparative study has established that light-activated UDMA resin is a more efficient material for provisional dental restorations when considering chair time and periodontal health. While chemically activated PMMA resin exhibits commendable handling properties; the advantages of UDMA in clinical settings are clear. The reduced chair time associated with UDMA not only improves patient comfort but also allows for enhanced clinical efficiency, which could potentially lead to increased patient throughput in busy dental practices. Moreover, the superior periodontal outcomes associated with UDMA resin suggest a reduced risk of gingival inflammation and plaque accumulation, contributing to better oral health during the provisional phase of treatment. This finding is particularly significant as it underscores the importance of material choice in the prevention of periodontal issues. In conclusion, our findings advocate for the consideration of light-activated UDMA resin as a preferred material for provisional restorations, balancing clinical efficiency with patient-centered outcomes. As the dental materials landscape continues to evolve, studies like ours play a crucial role in informing evidence-based clinical decisions that ultimately benefit patient care.
